# Integrated Laboratory Evaluation of Rift Valley Fever Virus Antibodies Using the Competitive ELISA and Virus Neutralization Test

**DOI:** 10.3390/pathogens15030264

**Published:** 2026-03-02

**Authors:** Ommer M. Dafalla, Abdullah A. Alashor, Mohammed O. Hussien, Elsiddig M. Noureldin, Tellal B. Ageep, Mohammed A. Najmi, Mohamed S. Mohamed, Ali A. Hakami, Saleh A. Alrashedi, Fisal A. Bushlaibi, Fahad N. Abukhalil

**Affiliations:** 1 Jazan Laboratory, General Department of Laboratories, Operation Sector, The National Center for the Prevention & Control of Plant Pests & Animal Diseases (Weqaa), Jizan 82224, Saudi Arabia; aalashor@weqaa.gov.sa (A.A.A.); moh200432@hotmail.com (M.O.H.); siddignoureldin@hotmail.com (E.M.N.); tellalageep92@gmail.com (T.B.A.); mhommadi@weqaa.gov.sa (M.A.N.); mohamedsami2233@gmail.com (M.S.M.); mlt.alihakami@gmail.com (A.A.H.); 2 General Department of Laboratories, Operation Sector, The National Center for the Prevention & Control of Plant Pests & Animal Diseases (Weqaa), Riyadh 11461, Saudi Arabia; salrashedi@weqaa.gov.sa (S.A.A.); fbushlaibi@weqaa.gov.sa (F.A.B.); fabukhalil@weqaa.gov.sa (F.N.A.)

**Keywords:** Rift Valley fever virus, Smithburn vaccine, virus neutralization test, competitive ELISA, *TCID*_50_, antibody response, vero cells, CPE, immunodiagnostic

## Abstract

Background: Rift Valley fever virus (RVFV) is a significant mosquito-borne zoonotic virus with high public health and veterinary importance in Africa and the Middle East. Reliable diagnostic assays for detecting antibodies and assessing their functional neutralizing capacity are essential for surveillance programs, vaccine monitoring, and outbreak preparedness. Objective: This study evaluates and compares the analytical performance of a competitive enzyme-linked immunosorbent assay (cELISA) and a virus neutralization test (VNT) for detecting RVFV antibodies in vaccinated sheep sera, establishing an integrated laboratory workflow for virus titration, serological detection, and functional neutralization. Methods: Twenty serum samples were collected from sheep pre-vaccination and one month post-vaccination with Smithburn live attenuated RVFV vaccine. Sera were tested using a commercial multispecies RVFV competitive ELISA to detect antibodies specific to the viral nucleocapsid protein. Viral titration was conducted in Vero cells, and 50% tissue culture infective dose (*TCID*_50_/0.1 mL) was calculated using the Reed and Muench method. VNT was performed at 24, 48, 72, and 96 h after infection with different viral doses (10^2^ to 10^5^ *TCID*_50_/0.1 mL), and the neutralizing ability of serial serum dilutions (1:2 to 1:1024) was tested. Compared with the control, protection was determined by cytopathic effect (CPE) inhibition. Results: ELISA revealed robust antibody signals up to a 1:32 dilution, with signal-to-noise (*S*/*N*) < 40%, whereas for higher dilutions, antibody detection became inconclusive or negative. Virus titration was performed to verify a stock concentration of 10^6.5^ *TCID*_50_/0.1 mL. The VNT exhibited time- and dose-dependent kinetics; high protection rates (≥97) were observed at 1:2–1:8 dilutions against 10^2^–10^3^ *TCID*_50_/0.1 mL challenge doses; however, neutralizing efficacy decreased significantly at higher viral loads and higher serum dilutions. While cELISA and VNT results correlated strongly at low serum dilutions, the cELISA showed decreased sensitivity at dilutions ≥ 1:64, where the VNT remained capable of detecting functional neutralizing activity. Conclusions/Discussion: The results demonstrate that while both assays correlate well at high antibody concentrations, they diverge at lower concentrations. This discrepancy highlights the functional difference between binding antibodies (N-protein) and neutralizing antibodies (Gn/Gc glycoproteins). Consequently, the cELISA is ideal for rapid screening, whereas the VNT is indispensable for confirming functional immunity. Integrating both assays provides a more accurate immunological profile for RVFV surveillance and vaccine evaluation.

## 1. Introduction

The Rift Valley fever virus (RVFV) is a zoonotic arthropod-borne virus of substantial public health and veterinary importance. It is classified under the genus *Phlebovirus* of the family *Phenuiviridae*. It was first isolated during an epidemic in the Rift Valley in Kenya in the early 1930s and subsequently caused recurrent epidemics throughout sub-Saharan Africa, the Arabian Peninsula and the Indian Ocean islands, usually accompanied by high mortality among young livestock and severe disease in humans [1,2,3]. Aedes and Culex mosquitoes are the primary vectors of the virus, although transmission can also occur among humans upon direct contact with the blood or organs of the infected animals [4]. In endemic regions, RVFV is mainly controlled through vector surveillance, early detection and vaccination of susceptible domestic animal species.

A live-attenuated vaccine (Smithburn strain) from the ancestral RVFV isolate is still in use because it induces strong immunogenic responses and long-lasting protection in animals [5]. Differences in viremia among vaccinated individuals may arise from variations in vaccine titers (during handling, transportation, or storage) and discrepancies in host antibody responses, which require the development of sensitive in vitro assays to assess the vaccine quality and immunological responses. Three important tools for assessing vaccine-induced immunity have become prominent: virus titration with *TCID*_50_, competitive enzyme-linked immunosorbent assays, and virus neutralization tests [6,7]. *TCID*_50_ determination, based on the Reed and Muench method, enables quantification of infectious virus and is fundamental for standardizing virus input in downstream neutralization assays [8]. Precise virus titration is critical, as VNT outcomes are highly sensitive to viral dose and antibody concentration [9].

The competitive ELISA is widely used for serological screening because of its high throughput, species independence, and suitability for field surveillance [10,11].

Nevertheless, although ELISAs are sensitive to antibody binding, they do not directly measure the neutralizing activity. In contrast, VNT is the gold standard for testing protective antibodies, as it directly measures the ability of serum antibodies to inhibit viral infectivity in cell culture, typically using highly permissive Vero cells [12]. Neutralization titers derived from VNT provide an important functional correlate of immunity and are commonly used to benchmark vaccine-induced antibody responses [13,14].

The combination of these three diagnostic methods (virus titration, ELISA and VNT) provided a complete enumeration of the immune response.

This study not only verifies the approach of assessing the RVFV vaccine but also has implications for virological diagnostics in general by illustrating the importance of combining assays based on antigen-binding and function. In the context of newly emerging zoonoses and increased focus on pandemic preparedness, sound testing principles for serological evaluation are becoming increasingly important. Furthermore, knowledge gained from RVFV studies could be applied to other arboviruses transmitted in a similar manner and provoke similar immune responses, such as the West Nile, Zika, and dengue viruses [4,15].

Ultimately, the objective of this study was to validate diagnostic methodologies and establish standardized laboratory protocols for RVFV serology and neutralization testing, and aimed to increase the precision of immunological assessments after RVFV vaccination and provide a methodological approach to combine viral quantification, serological detection and functional neutralization testing. This type of analysis is necessary to confirm vaccine effectiveness and to understand the kinetics of antibodies and correlates of protection, especially in low-resource settings where RVFV is endemic.

## 2. Materials and Methods

### 2.1. Virus Dilution Preparation for Virus Titration

The Rift Valley fever live-attenuated vaccine (Smithburn strain, Batch No. 136, Onderstepoort, Pretoria, South Africa) was provided by the Rift Valley Fever Control Program, Weqaa Center, Jazan region. The lyophilized vaccine was reconstituted and ten-fold serially diluted in Roswell Park Memorial Institute medium, Thermo Fisher Scientific (Gibco), New York, NY, USA (RPMI 1640). Dilutions were prepared in nine 3 mL tubes containing 1.8 mL of RPMI media supplemented with 1% antibiotic antimycotic and 0% fetal bovine serum (FBS). Briefly, 0.2 mL of the reconstituted vaccine was added to the first tube; after thorough vortexing, 0.2 mL was sequentially transferred to each subsequent tube to achieve the desired ten-fold dilution series.

### 2.2. Cells and Medium

Vero cells (ATCC^®^ CCL-81™) were cultured in 75 cm^2^ culture flasks (Nest Biotech, Wuxi, China) using RPMI 1640 medium supplemented with 10% FBS (Gibco, New York, NY, USA) and 1% penicillin–streptomycin (Thermo Scientific, New York, NY, USA) and incubated at 37 °C in a 5% CO_2_ incubator.

### 2.3. RVF Virus Titration

To verify the infectivity of the vaccine stock following transportation and storage, local titration was performed prior to the virus neutralization test (VNT). The viral titer was determined by calculating the tissue culture infectious dose 50% (*TCID*_50_) using the Reed and Muench method [8].

Vero cells were trypsinized, and viable cell density was determined using a hemocytometer [16] via the trypan blue exclusion method (100 μL cell suspension to 100 μL dye). The cells were suspended at a concentration of 2 × 10^4^ cells/mL in RPMI medium supplemented with 10% FBS + 2% antibiotic/antimycotic. The 96-well tissue culture plate was seeded with 100 μL of cell suspension per well and incubated for 24 h until the monolayer reached 90% confluence.

Following incubation, the medium was aspirated, and the Vero cell monolayers were washed with phosphate-buffered saline (PBS). Subsequently, 100 µL of each viral dilution was inoculated into six replicate wells (Rows B–G; columns 2–10), while Column 11 served as the negative control (Table 1). After 37 °C and 5% CO_2_ incubation for one hour to allow viral adsorption, 100 µL of maintenance medium (RPMI with 2% FBS and 2% antibiotic/antimycotic) was added to each well. Peripheral wells were filled with 200 µL of PBS to create a humidity chamber and prevent evaporation in the inoculated wells. The plate was subsequently incubated and observed daily using an inverted microscope for cytopathic effects (CPEs) characterized by cell rounding, detachment, and clumping. The incubation period was determined by the progression of CBEs, reaching the endpoint (defined as >90% CPEs at the highest virus concentration) after four days.

Mathematical Calculation: The *TCID*_50_ was calculated using the method suggested by Reed and Muench [8] according to the following formula:

log10 *TCID*_50_ = log10 (dilution above 50%) + (*PD* × log10 dilution factor).

The infection rate was calculated as follows:

Infection rate = [cumulative positive units/(cumulative positive units + cumulative negative units)] × 100.

The interpolated value of the 50% endpoint (I), also known as the proportional distance (*PD*):

*PD* = (% positive at dilution above 50% − 50%)/(% positive at dilution above 50% − % positive at dilution below 50%).

Since each well was inoculated with 0.1 mL of the virus dilution, the resulting titer was initially expressed as *TCID*_50_/0.1 mL.

### 2.4. Samples Collection

As part of the national RVF surveillance program in the Jazan region of southwest Saudi Arabia, twenty sheep were selected from herds monitored under the supervision of field veterinarians (WEQAA Center, Jazan, Saudi Arabia). To confirm the absence of RVFV antibodies, serum samples were collected from all animals prior to vaccination (pre-vaccination). The animals were then vaccinated with the RVF Smithburn live-attenuated vaccine. The stock vaccine titer 10^6.5^ *TCID*_50_/0.1 mL) was reconstituted and diluted to a final administrative concentration of 10^4.5^ *TCID*_50_ per dose.

One month post-vaccination, second serum samples were collected from the same twenty animals. For each collection, approximately 5 mL of blood was collected aseptically from the jugular vein of each animal using sterile disposable needles and vacutainer tubes without anticoagulant, allowed to clot at room temperature, and centrifuged at 3000 rpm for 10 min. Sera were aliquoted and stored at −20 °C until analysis.

No anesthesia was administered prior to blood collection, as the procedure was minimally invasive and consistent with routine veterinary practice for sample collection in livestock.

### 2.5. Preparation of RVF Antibody Dilutions for Competitive ELISA and VNT

Initially, the collected serum samples were screened for the presence of RVF antibodies using the ID Screen Rift Valley Fever Competition Multispecies Kit (IDvet, Grabels, France). Ten strongly positive samples with optical density (O.D) ranging from 0.05 to 0.06 were pooled and filtered through a 0.22 μm syringe filter. The pooled serum was inactivated by heating at 56 °C for 1 h to complement inactivate the samples, and 10 serial two-fold dilutions were prepared in RPMI 1640 (with 1% antibiotic antimycotic, without serum) as follows: 1:2, 1:4, 1:8, 1:16, 1:32, 1:64, 1:128, 1:256, 1:512, and 1:1024. Fetal bovine serum (FBS) was similarly diluted and served as a negative control.

### 2.6. Competitive ELISA

A Competition Multispecies Kit (Innovative Diagnostic, IDvet, Grabels, France) was used according to the manufacturer’s instructions. Each serum dilution (1:2 to 1:1024) was tested in triplicate. The assay plate includes the manufacturer-provided negative and positive controls, alongside additional controls prepared in-house: two negative controls (FBS), and two concentrated positive control samples. For each sample, the competition percentage, or signal-to-noise ratio (*S*/*N*%), was calculated using the following formula:

*S*/*N*% = (O.D sample/O.D negative) × 100.

According to the manufacturer’s diagnosis criteria, the results were interpreted as follows:

- Positive: *S*/*N*% ≤ 40%.
- Doubtful: 40% < *S*/*N*% ≤ 50%.
- Negative: *S*/*N*% > 50%.

### 2.7. Preparation of Virus Dilutions for VNT

To assess the neutralization capacity of serum antibodies against RVFV infectivity, the VNT assay was performed as previously described, with slight modifications [6,17,18].

Upon verification of the stock titer (10^6.5^/0.1 mL), the vaccine was standardized to a working concentration of 10^6^/0.1 mL using the *C*1 × *V*1 = *C*2 × *V*2 formula to obtain a solution with a concentration of 10^−6^. Briefly, 1 mL of the reconstituted vaccine stock (*C*1 = 10^6.5^) was diluted to a final volume (*V*2) of 3.16 mL (*C*2 = 10^6^), using serum-free RPMI medium.

From this standardized working stock, ten-fold serial dilutions were prepared. For each dilution step, 1.8 mL of RPMI medium (supplemented with 1% antibiotic–antimycotic and 0% FBS) was aliquoted into 3 mL tubes. Subsequently, 0.2 mL of the virus preparation was added to the first tube; following thorough vortexing, 0.2 mL was sequentially transferred to the next tube to achieve the desired dilution series (Table 2).

### 2.8. Virus Neutralization Test and NT_50_ Determination

The virus neutralization test (VNT) was performed using Vero cell monolayers to evaluate the inhibitory capacity of immune serum against RVFV.

Experimental Procedure: Serial two-fold dilutions of pooled immune serum (1:2 to 1:1024) were prepared and mixed with RVFV challenge doses ranging from 100 to 100,000 *TCID*_50_/0.1 mL. For each virus concentration, 250 μL of the viral inoculum was combined with 250 μL of either the positive or negative serum dilution in 1.5 mL tubes. These mixtures (500 μL total volume) were incubated at 37 °C for one hour to allow for neutralization. Following incubation, 100 μL of each virus–serum mixture was inoculated into 4-well replicates of Vero cell monolayers. The plate layout (detailed in Table 3) utilized columns 2–11 for virus–serum mixtures, with the top half (rows A–D) receiving positive serum and the bottom half (rows E–H) receiving negative serum. Column 1 served as the positive virus control (virus without serum), while column 12 served as the cell control (medium only) to monitor cell health.

Quantification and Endpoints: Inoculated cells were monitored for cytopathic effects (CPEs) for up to 96 h post-infection. Neutralization activity was calculated as the percentage of CPE inhibition relative to the virus-only controls. The 50% neutralization titer (*NT*_50_) was defined as the highest serum dilution, resulting in ≥50% reduction in CPEs for a specific virus challenge dose. *NT*_50_ endpoints were determined through a combination of visual inspection and quantitative assessment of CPE inhibition patterns.

For example, in Plate 1, wells A2 to D2 were inoculated with the virus dilution 10^−1^ and 1:2 antibody mixture, whereas wells E2 to H2 were inoculated with the virus 10^−1^ and 1:2 negative antibody serum mixtures. To assess and calculate the difference in the cytopathic effects (CPEs) between virus dilutions incubated with antibodies and those mixed with negative control serum, each plate (A2 to D2) and columns 2–11 were inoculated with a virus-positive antibody mixture, whereas E2–H2 and columns 2–11 were inoculated with virus-negative antibody serum mixtures. Each plate included a positive control (virus concentration without serum) in column 1 and a control (cells with medium) in column 12 to assess cell quality. The same experimental design was applied to Plates 2, 3, and 4 to evaluate the neutralization capacity against the subsequent virus dilutions.

### 2.9. Calculation of the Protection Rate

The protection rate (%) was used to quantify how effectively the antibody serum prevented viral CPEs in cell culture using the following formula: protection rate % = (1 − CPE mean of treated/CPE mean of virus control) × 100, where CPE mean of virus control = 100 [8,12,19]. If the mean CPE of the control was not 100, we used the actual value in the following formula: protection rate = ((control CPE mean–treated CPE mean)/control CPE mean)) × 100.

The neutralization titer (NT) was calculated as follows:

A 50% neutralization titer is defined as the reciprocal of the highest dilution of the antibody that induces a 50% reduction in CPEs by the virus in vitro [13,20,21,22,23]. It is often employed in plaque reduction neutralization tests (PRNT) or microneutralization tests to quantify neutralizing antibody titers.

### 2.10. Assay Validation and Interpretation

The positive control (virus with no serum) produced 100% CPEs, and the negative control (cells with no virus) produced 0% CPEs at the end of each test; thus, testing the assay negativity could proceed to calculate the endpoint of the titration test.

The neutralizing titer can be defined as the reciprocal of the highest dilution that results in ≥50% protection (e.g., 1:8 at 100,000 *TCID*_50_/mL) [24].

The variation in the number of positive wells and CPEs range indicates some experimental variability, which is common in VNTs and supports the need for replicates and statistical confidence in reported titers [25].

### 2.11. Statistical Analysis

Statistical packages for social sciences (SPSS ver. 25) and Microsoft Excel were used for data manipulation, analysis and visualization.

The CBE rate (%) and protection rate (%) were calculated using Microsoft Excel. Spearman’s correlation was used to examine the correlation between antibody dilution and CPE, and the test was considered significant when the *p*-value was less than 0.05.

## 3. Study Results

### 3.1. RVF Competitive ELISA Results

According to the manufacturer’s interpretation criteria, all twenty pre-vaccination samples were found to be negative for RVFV antibodies, whereas all twenty post-vaccination samples were positive.

In the serial double-fold analysis of pooled positive sera, antibody detection was heavily identified at serum dilutions of 1:2–1:32, as represented in Table 4, with *S*/*N* (3–23%) representing positivity. The 1:64 dilution resulted in *S*/*N* values ranging between 41% and 47% and was categorized as doubtful. From dilutions 1:128 and above, the *S*/*N*% values were greater than 50%; hence, they were considered negative.

**Figure 1:**
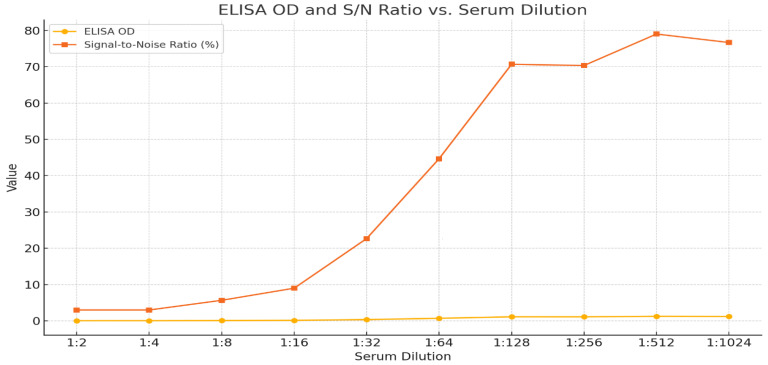
ELISA optical density values and signal-to-noise ratios at serial serum dilutions (1:2–1:1024).

The graphical plot (Figure 1) shows the O.D values and corresponding *S*/*N* ratios, which reflect the level of antibodies that bind to the viral antigen. A gradual increase in both parameters with increasing dilutions indicated a strong antibody response.

### 3.2. Observations of CPE

On virus titration and VNT plates, cytopathic effects (CPEs) were examined at 100× magnification using an inverted microscope at 96 h post-incubation.

**Figure 2:**
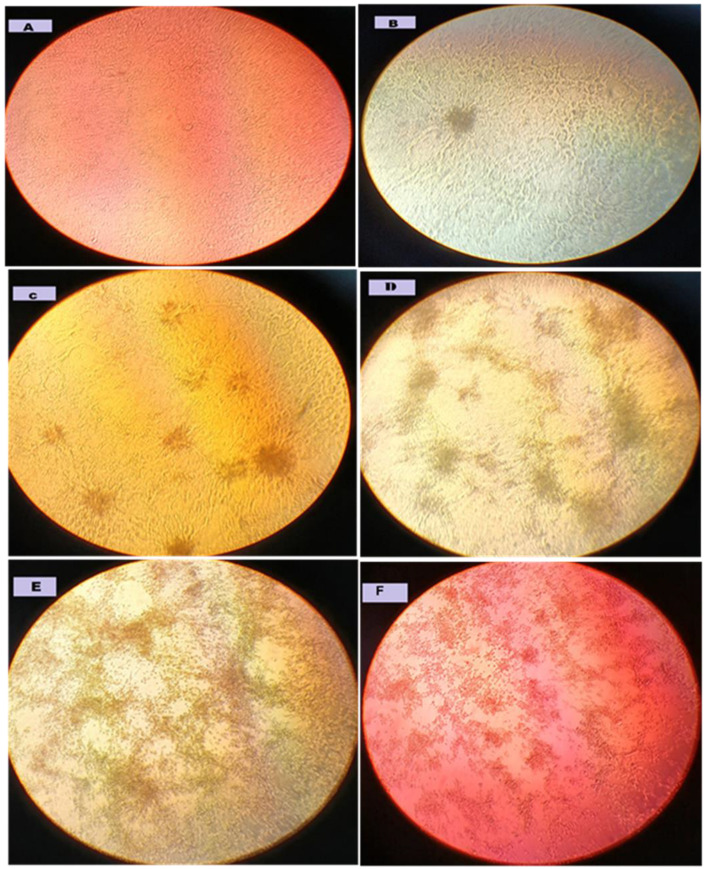
Temporal stages of CPE development in RVFV-infected Vero cells. (**A**) Normal uninfected Vero cells. (**B**) Initial low-level CPE: 1–10% CPE. (**C**) Moderate CPE with enhanced cell rounding of 30–50% CPE. (**D**) Advanced CPE with 60–70% CPE. (**E**) Destruction of the monolayer with massive cell lysis and 90% CPE. (**F**) Full CPE, with most cells detached or lysed (approx. 100% CPE).

Figure 2 shows the dynamics of CPE development in RVFV-infected Vero cells, from a normal appearance (control) to total monolayer damage. The main findings of each stage are as follows:

(A) Normal Vero cells, non-infected culture with a healthy monolayer, flat, elongated, and in close contact. (B) Low-level CPEs (loose clusters of mildly rounded cells, sometimes detached) and later sporadic CPEs (1–10%). (C) Moderate CPEs with increased cell rounding, granulation, and focal monolayer disruption (30–50% CPE). Rounded or detached cells clustered into large foci, predominantly in darker areas (400× magnification). These may be early plaques or CPE foci. (D) Late CPEs with scattered anchorage-dependent cells with cytoplasmic condensation and released interstitial-like cells (70% CPE). Note the well-defined empty spaces where cells have been released, and the adherent cells appear disorganized and frequently clustered. (E), (F) Destruction of the monolayer with massive cell lysis (90% CPE) or full 100% CPE, desorption with most cells detached or lysed, severe CPEs with complete monolayer destruction, there was high virus activity and/or inadequate neutralization by antibody.

### 3.3. Virus Titration and TCID_50_ Calculation Results

A viral titration experiment was conducted to determine the infectious titer of the RVF vaccine. Ten-fold serial dilutions ranging from 10^−1^ to 10^−9^ were applied to Vero cell cultures, with six replicate wells for each dilution. The cytopathic effects (CPEs) were monitored daily. After 96 h of incubation, 100% CPE was observed in the wells from dilutions of 10^−1^ to 10^−4^. Partial CPE appeared at a dilution of 10^−5^, and the infection rate decreased markedly at dilutions of 10^−7^ or higher. Table 5 presents the percentage of observed CPEs, cumulative positive and negative wells, and calculated infection rates across serial dilutions.

To estimate the *TCID*_50_, the Reed and Muench method was used according to the following formula: log10 *TCID*_50_ = log10 (dilution above 50%) + (*PD* × log10 dilution factor).

For proportional distance (*PD*), we used the following formula: (% positive at dilution above 50% − 50%)/(% positive at dilution above 50% − % positive at dilution below 50%). Then, we applied the following formula: *PD* = (50 − 70/70 − 50) = −0.5.

*TCID*_50_ calculation: Since the dilution factor is 10 (ten-fold), the log of the dilution factor is 1.

log10 *TCID*_50_ = log10 (dilution above 50%) + (*PD* × log10 dilution factor).

log10 *TCID*_50_ = −6 + (−0.5 × 1) = −6.5. The titer is 10^−6.5^/0.1 mL.

Notably, our results were consistent with the titer provided by the vaccine producer, which was 10^6.5^.

### 3.4. Virus Neutralization Test (VNT) Results and NT_50_ Determination

**Figure 3:**
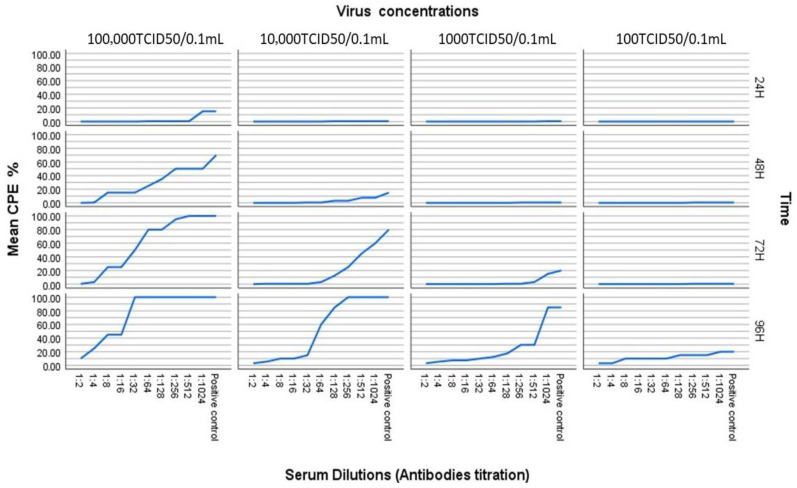
VNT results showing CPE inhibition across varying antibody dilutions and the CPE range, mean percentage for each condition.

The neutralizing capacity of RVFV antibodies was assessed across a range of serum dilutions (1:2–1:1024) and virus challenge doses (10^2^–10^5^ *TCID*_50_/0.1 mL). Neutralization was evaluated based on the inhibition of CPEs in Vero cell monolayers over a 96 h observation period (Figure 3 and Table S1).

At the highest virus concentration (100,000 *TCID*_50_/0.1 mL), rapid and widespread CPE development was observed across nearly all antibody dilutions. By 96 h, the CPE reached approximately 45% at a 1:8 dilution, and progressed to 100% from 1:16 onwards. These results indicate that only the lowest dilutions (highly concentrated antibodies, 1:2 and 1:4) maintained high protection rates of 90% and 75%, respectively.

At an intermediate virus challenge dose (10,000 *TCID*_50_/0.1 mL), antibody-mediated protection was better preserved at lower serum dilutions. At 96 h post-infection, CPEs ranged from 3% at 1:2 dilution to 100% at dilutions ≥ 1:128. Protection rates remained ≥85% up to a dilution of 1:16, whereas higher dilutions resulted in substantial CPEs due to insufficient antibody concentration.

Effective neutralization was most pronounced at low virus concentrations (1000 and 100 *TCID*_50_/0.1 mL). At these loads, protection remained above 50% even at a 1:512 dilution at 96 h post-infection. These findings support the conclusion that lower viral loads are more effectively controlled by pre-existing antibodies, and align with established correlates of protection in an in vivo model.

### 3.5. NT_50_ Endpoints and Correlation with Antibody Dilution

Based on the ≥50% CPE inhibition threshold, *NT*_50_ values shifted significantly according to the virus challenge dose. At low virus concentrations (100–1000 *TCID*_50_/0.1 mL), *NT*_50_ endpoints were observed at serum dilutions ranging from 1:128 to 1:512, whereas at intermediate virus doses (10,000 *TCID*_50_/0.1 mL), *NT*_50_ shifted toward a lower dilution of approximately 1:16. At the highest challenge dose (100,000 *TCID*_50_/0.1 mL), ≥50% neutralization was achieved only at the lowest serum dilutions (≤1:4), confirming a strong dose-dependent reduction in neutralization efficiency.

Spearman’s correlation analysis demonstrated a significant inverse correlation between serum dilution and CPE development (*p* < 0.05). The rho coefficients (*p*) were 0.813, 0.969, 0.994, and 0.879 for virus doses of 100,000, 10,000, 1000, and 100 *TCID*_50_/0.1 mL, respectively. These high coefficients confirm that higher antibody concentrations are strictly associated with increased neutralization capacity.

### 3.6. Time-Dependent Effect on CPE

**Figure 4:**
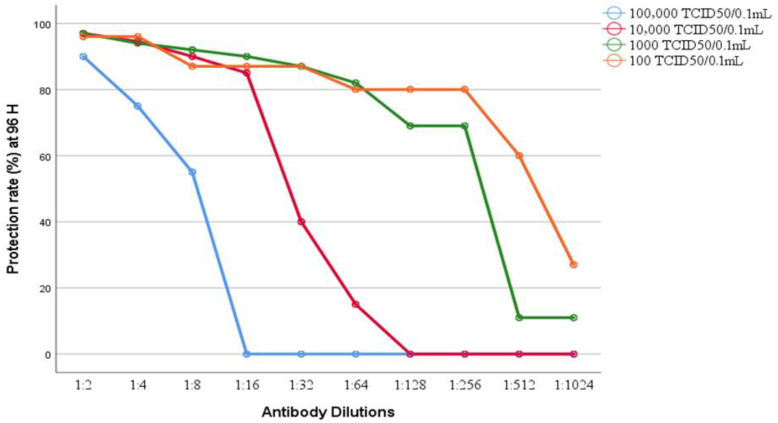
Protection rate (%) against different serum antibody dilutions at 96 hpost-infection for various virus concentrations.

Cytopathic effect (CPE) development exhibited a clear time-dependent progression across all experimental conditions, with median CPE values increasing significantly from 24 to 96 h post-infection. Early-onset CPE was recorded as early as 48 h in wells combining high viral load and low serum concentration. These results highlight the synergistic influence of virus dose, antibody concentration, and incubation duration on neutralization outcomes. Table 6 and Figure 4 emphasize that robust neutralization is maintained longest at lower dilution and reduced virus concentration.

To support and contextualize our VNT findings according to the references for CPEs and neutralization assay standards [5,9,13,14,26], Table 7 shows a comparison of the results with standard or expected outcomes in viral neutralization assays, especially those measuring CPEs as an endpoint. Table 8 presents the observations, interpretations, and potential explanations. The comparison results revealed that our VNT findings align very well with standard viral neutralization patterns, the serum contains potent antibodies, especially those against lower viral loads, and the assay functions as expected, showing clear dose-dependent antibody–virus interactions.

The expected results of a strong neutralization response were a low CPE at a low antibody dilution (high concentration) and a gradual increase in CPEs as the antibody concentration decreased. At high dilutions (1:128, 1:256, etc.), CPE approached 100%, indicating a loss of neutralization.

## 4. Discussion

Reliable laboratory diagnosis of Rift Valley fever virus (RVFV) is essential for surveillance programs, vaccine monitoring, and outbreak preparedness in endemic regions. This study presents an integrated laboratory evaluation of RVFV antibody detection using competitive ELISA and virus neutralization testing (VNT), supported by standardized virus titration using the Reed–Muench *TCID*_50_ method.

The competitive ELISA demonstrated robust analytical performance for antibody detection at low and moderate serum dilutions (1:2–1:32), consistent with previous reports validating nucleoprotein-based RVFV ELISAs for multispecies surveillance. At higher dilutions (≥1:64), the assay sensitivity declined, resulting in doubtful or negative results. This observation is consistent with the known limitation of binding assays, which detect physical antigen–antibody interaction but do not necessarily reflect functional virus neutralization. The observed discrepancy at high dilutions likely stems from fundamental differences in the antibodies these assays detect; while the cELISA primarily targets the highly immunogenic nucleocapsid (N) protein, the VNT is restricted to antibodies targeting neutralizing epitopes on surface glycoproteins, Gn and Gc [27]. Furthermore, at these higher dilutions, the cELISA may fail to detect low-affinity antibodies that no longer meet the binding threshold of the assay, whereas the VNT measures a functional biological outcome [28].

These findings are in line with the established threshold guidelines for competitive ELISAs [29] and are consistent with previous studies that demonstrated the detection of antibodies as early as a 1:32 dilution after RVFV exposure [27,30]. Moreover, the gradual increase in *S*/*N*% and O.D observed upon further dilution is representative of well-established antibody-binding kinetics: high-affinity binding at high concentrations followed by predictable signal decay at the antibody concentration decreases [11]. Paweska et al. [10] reported comparable results, supporting the suitability of competitive ELISA for field surveillance and vaccine response assessment.

A high-dose vaccine virus stock was confirmed at atiter of 10^6.5^ *TCID*_50_/0.1 mL, using the Reed and Muench method. Consistent with the expected outcomes for live-attenuated vaccines, the precision of this infectious dose was validated by the observation of 100% CPE at dilutions up to 10^−4^ and the 50% endpoint at approximately 10^−6.5^ [8,31]. This aligns with the manufacturer specifications and confirms the high-titer reproducible inoculum required for rigorous neutralization studies [15].

In contrast, the VNT results provided a clear inverse correlation between serum dilution and viral CPE, with substantial protection (>90%) at dilutions of 1:2 and 1:4 across all tested virus concentrations. As both the virus challenge dose and serum dilution increased, the level of protection gradually decreased. This dose-dependent neutralization pattern is consistent with previous findings in CPE-based neutralization assays [9,26], where the viral input directly influences the perceived neutralizing capacity of the serum.

At high viral loads (10^5^ *TCID*_50_/0.1 mL), protective efficacy was restricted to the lowest dilutions (1:2–1:4), whereas at intermediate viral loads (10^4^ *TCID*_50_/0.1 mL), protection was extended to a 1:16 dilution. Functional neutralization was detectable up to a 1:256 dilution, specifically at lower virus titers (10^3^ and 10^2^ *TCID*_50_/0.1 mL). These results are in agreement with those of Manenti et al. [7] and Muruato et al. [14], who reported that lower viral challenge doses result in more durable and detectable neutralization profiles.

Cellular changes during CPE development followed a clear time-dependent manner between 24 and 96 h post-infection. Under conditions of high-viral input and low antibody concentration, early signs of infection were evident by 48 h, and by 96 h, the majority of high-dilution groups exhibited complete CPE. This finding is consistent with previous reports that the level of viral replication is associated with the extent of CPE development over time [12,32] and confirms the replication kinetics of RVFV in Vero cells.

A strong correlation between VNT activity at low serum dilutions and competitive ELISA positivity indicates that both assays are suitable for detecting RVFV antibodies during early immune responses. At higher dilutions (≥1:64), ELISA sensitivity declined, whereas VNT continued to detect neutralizing activity, reflecting fundamental differences between binding- and function-based assays. This divergence underscores the non-linear relationship between total binding antibody concentration and neutralization capacity; a high concentration of binding antibodies does not always equate to a proportional increase in neutralization, as the latter requires a specific threshold of epitope occupancy and “critical mass” of high-affinity antibodies to effectively inhibit viral entry [33].

While ELISA measures antibody–antigen binding, VNT directly quantifies functional virus neutralization, which may better reflect protective immunity, as previously reported [13].

Our results are also consistent with the neutralization and ELISA benchmark values reported in previous studies [4,5,34]. The Reed–Muench method is still a reference technique for *TCID*_50_ estimation and has been widely applied in flavivirus and bunyavirus research. Additionally, the time-resolved protective profile of the VNT and the good precision of the ELISA test at low dilutions indicate that both methods are potentially valuable tools for the evaluation of antibodies.

Crucially, our findings are consistent with the concept of ‘dilutional loss of protection’, which has been previously observed in RVFV and related arbovirus challenge models [1,2]. The VNT results provide a reliable measure of functional immunity, especially for low viral loads, despite the decrease in ELISA reactivity at high dilutions.

The findings of this study have direct implications for Rift Valley fever virus (RVFV) surveillance and diagnostic workflows in endemic and at-risk regions. Competitive ELISA demonstrated high sensitivity for detecting RVFV antibodies at low serum dilutions, supporting its suitability as a first-line, high-throughput screening tool in large-scale field surveillance programs. This is particularly relevant for routine monitoring of livestock populations, where rapid processing of large numbers of samples is required.

Conversely, the VNT provided functional confirmation of antibody activity and enabled quantitative assessment of neutralizing titers across serial serum dilutions. These characteristics make VNT particularly valuable as a confirmatory assay in reference laboratories, especially in vaccinated populations where differentiation between seropositivity and functional neutralization is critical. The observed decline in antibody detection at higher dilutions further highlights the complementary nature of ELISA and VNT, emphasizing that reliance on a single serological assay may lead to under- or over-estimation of RVFV exposure.

In conclusion, the combined application of competitive ELISA for initial screening and VNT for confirmatory and functional assessment offers a robust and practical diagnostic strategy for RVF surveillance programs. This integrated approach supports accurate evaluation of RVFV exposure, enhances confidence in serological interpretation, and aligns with the diagnostic needs of national surveillance and reference laboratory systems

## 5. Study Conclusions

The complementary nature of ELISA and VNT is well illustrated by the present data. While competitive ELISA is suitable for large-scale serological screening due to its speed and scalability, VNT remains the gold standard for confirming protective immunity. The combined diagnostic workflow provides a robust laboratory framework for RVFV antibody profiling and supports integrated surveillance strategies in endemic regions.

Our findings emphasize that neutralizing efficacy is highly dependent on both the antibody concentration and the viral challenge dose. The observed “dilutional loss of protection” highlights a critical threshold in immune monitoring: a negative ELISA result does not necessarily equate to a lack of protective immunity, particularly against lower viral loads.

Importantly, this study was designed as a laboratory-based diagnostic evaluation using field sera collected under a national vaccination program. The objective was not to assess vaccine efficacy or protection in vivo, but rather to validate diagnostic methodologies and establish standardized laboratory protocols for RVFV serology and neutralization testing.

## 6. Limitations of the Study

The primary focus of this study was a controlled laboratory evaluation of VNT dynamics rather than a population-level vaccine assessment. To ensure a standardized antibody source and minimize inter-individual variability, pooled sera from seropositive animals were utilized. While this approach enabled a precise comparison of CPE inhibition across multiple viral challenge doses, it limits the extrapolation of findings regarding individual immune variability and diagnostic sensitivity across diverse field samples.

Additionally, outcomes were expressed as CPE inhibition patterns and protection rates rather than formal *NT*50 calculations for every combination. While these trends align with established VNT behavior, future work incorporating individual titers and automated quantification could further enhance the analytical depth of this methodology. Finally, the original complex data visualizations have been moved to the Supplementary Material to prioritize clarity while maintaining full transparency.

## Figures and Tables

**Table 1:** Layout of the microtiter plate, indicating distribution of serial dilutions of the test virus (10^−1^ to 10^−9^), PBS, and negative controls (−Ve).

	1	2	3	4	5	6	7	8	9	10	11	12
**A**	PBS	PBS	PBS	PBS	PBS	PBS	PBS	PBS	PBS	PBS	PBS	PBS
**B**	PBS	10^−1^	10^−2^	10^−3^	10^−4^	10^−5^	10^−6^	10^−7^	10^−8^	10^−9^	−Ve	PBS
**C**	PBS	10^−1^	10^−2^	10^−3^	10^−4^	10^−5^	10^−6^	10^−7^	10^−8^	10^−9^	−Ve	PBS
**D**	PBS	10^−1^	10^−2^	10^−3^	10^−4^	10^−5^	10^−6^	10^−7^	10^−8^	10^−9^	−Ve	PBS
**E**	PBS	10^−1^	10^−2^	10^−3^	10^−4^	10^−5^	10^−6^	10^−7^	10^−8^	10^−9^	−Ve	PBS
**F**	PBS	10^−1^	10^−2^	10^−3^	10^−4^	10^−5^	10^−6^	10^−7^	10^−8^	10^−9^	−Ve	PBS
**G**	PBS	10^−1^	10^−2^	10^−3^	10^−4^	10^−5^	10^−6^	10^−7^	10^−8^	10^−9^	−Ve	PBS
**H**	PBS	PBS	PBS	PBS	PBS	PBS	PBS	PBS	PBS	PBS	PBS	PBS

**Table 2:** Prepared virus dilutions used in the VNT.

Tube No.	Dilution	*TCID*_50_\0.1 mL
Tube 1	10^−1^	100,000 (10^5^)
Tube 2	10^−2^	10,000 (10^4^)
Tube 3	10^−3^	1000 (10^3^)
Tube 4	10^−4^	100 (10^2^)

**Table 3:** Plate 1, representative 96-well plate layout for RVFV neutralization (Plate 1: 10^−1^ virus dilution).

	Virus Dilution	RVF Antibodies Dilutions											
		1	2	3	4	5	6	7	8	9	10	11	12
		+C	1:2	1:4	1:8	1:16	1:32	1:64	1:128	1:256	1:512	1:1024	−C
**A**	10^−1^	10^−1^	10^−1^	10^−1^	10^−1^	10^−1^	10^−1^	10^−1^	10^−1^	10^−1^	10^−1^	10^−1^	−C
**B**		10^−1^	10^−1^	10^−1^	10^−1^	10^−1^	10^−1^	10^−1^	10^−1^	10^−1^	10^−1^	10^−1^	−C
**C**		10^−1^	10^−1^	10^−1^	10^−1^	10^−1^	10^−1^	10^−1^	10^−1^	10^−1^	10^−1^	10^−1^	−C
**D**		10^−1^	10^−1^	10^−1^	10^−1^	10^−1^	10^−1^	10^−1^	10^−1^	10^−1^	10^−1^	10^−1^	−C
**Negative FBS**		+C	1:2	1:4	1:8	1:16	1:32	1:64	1:128	1:256	1:512	1:1024	−C
**E**	10^−1^	10^−1^	10^−1^	10^−1^	10^−1^	10^−1^	10^−1^	10^−1^	10^−1^	10^−1^	10^−1^	10^−1^	−C
**F**		10^−1^	10^−1^	10^−1^	10^−1^	10^−1^	10^−1^	10^−1^	10^−1^	10^−1^	10^−1^	10^−1^	−C
**G**		10^−1^	10^−1^	10^−1^	10^−1^	10^−1^	10^−1^	10^−1^	10^−1^	10^−1^	10^−1^	10^−1^	−C
**H**		10^−1^	10^−1^	10^−1^	10^−1^	10^−1^	10^−1^	10^−1^	10^−1^	10^−1^	10^−1^	10^−1^	−C

**Table 4:** Signal-to-noise percentages (*S*/*N*%) obtained from three replicate wells at various serum dilutions.

Serum Antibodies Dilutions														
	Kit − Ve C	Kit + Ve C	FBS	+Ve C	1:2	1:4	1:8	1:16	1:32	1:64	1:128	1:256	1:512	1:1024
Well 1 OD	1.565	0.049	1.593	0.044	0.054	0.053	0.092	0.145	0.365	0.756	1.204	1.277	1.283	1.177
Well 1 *S*/*N*	98%	3%	100%	3%	3%	3%	6%	9%	23%	47%	76%	80%	81%	74%
Well 2 OD	1.62	0.055	1.594	0.047	0.051	0.054	0.087	0.158	0.362	0.726	1.063	1.015	1.248	1.216
Well 2 *S*/*N*	102%	3%	100%	3%	3%	3%	5%	10%	23%	46%	67%	64%	78%	76%
Well 3 OD					0.042	0.053	0.101	0.128	0.352	0.652	1.097	1.060	1.241	1.267
Well 3 *S*/*N*					3%	3%	6%	8%	22%	41%	69%	67%	78%	80%
Mean *S*/*N*	100%	3%	100%	3%	3%	3%	6%	9%	23%	45%	71%	70%	79%	77%
Results	Negative	Positive	Negative	Positive	Positive	Positive	Positive	Positive	Positive	Doubtful	Negative	Negative	Negative	Negative

**Table 5:** Infection rate (%) across 10-fold dilutions of virus.

Dilution	Observed CPE			Cumulative			Infection Rate A/(A + B)
	Total Well	Positive	Negative	Positive (A)	Negative (B)	Total A + B	
**10^−1^**	6	6	0	36 ↑	0 ↓	36	100
**10^−2^**	6	6	0	30 ↑	0 ↓	30	100
**10^−3^**	6	6	0	24 ↑	0 ↓	24	100
**10^−4^**	6	6	0	18 ↑	0 ↓	18	100
**10^−5^**	6	5	1	12 ↑	1 ↓	13	92
**10^−6^**	6	4	2	7 ↑	3 ↓	10	70
**10^−7^**	6	2	4	3 ↑	7 ↓	10	30
**10^−8^**	6	1	5	1 ↑	12 ↓	13	7.7
**10^−9^**	6	0	6	0 ↑	18 ↓	18	00

↑ (Up arrow) Indicates the cumulative number of positive wells (A); ↓ (Down arrow) Indicates the cumulative number of negative wells (B).

**Table 6:** Protection rate (%) at 96 h and ELISA results.

Antibodies Dilution	100,000 *TCID*_50_/0.1 mL	10,000 *TCID*_50_/0.1 mL	1000 *TCID*_50_/0.1 mL	100 *TCID*_50_/0.1 mL	Elisa Results
	Protection Rate%	Protection Rate%	Protection Rate%	Protection Rate%	
1:2	90	97	97	96	Positive
1:4	75	94.5	94	96	Positive
1:8	55	90	92	87	Positive
1:16	0	85	90	87	Positive
1:32	0	40	87	87	Positive
1:64	0	15	82	80	Negative
1:128	0	0	69	80	Negative
1:256	0	0	69	80	Negative
1:512	0	0	11	60	Negative
1:1024	0	0	11	27	Negative

**Table 7:** Comparison of VNT findings with standard or expected outcomes in viral neutralization assays.

Criterion	Study Results	Standard Expectation	Match?
Low CPE at 1:2, 1:4 for low virus titer	CPE ~3% for 100 & 1000 *TCID*_50_/0.1 mL	Yes—strong neutralization	yes
Rising CPE with dilution	CPE rises from 1:2 to 1:512	Expected as antibodies dilute	yes
High virus titers show 100% CPE even at 1:8–1:16	True for 100,000 *TCID*_50_/0.1 mL	Suggests overwhelming infection	yes
Positive control (no serum) shows 100% CPE	All virus doses show 100%, except 100 *TCID*_50_/0.1 mL show 80%	Confirms assay integrity	yes

**Table 8:** VNT study observations, interpretation, and potential explanations.

Observation	Interpretation	Potential Explanation
10,000 *TCID*_50_/0.1 mL still shows neutralization at 1:32 (CPE ~60%)	Slightly stronger than typical	Suggests serum has potent antibodies
1000 *TCID*_50_/0.1 mL shows CPE only ~30% up to 1:128 dilution	Mild CPE at moderate dilution	Indicates good neutralizing titer
100 *TCID*_50_/0.1 mL shows CPE ~65% even at 1:1024	Slight CPE at high dilutions is normal	Low virus amounts allow longer antibody protection

## Data Availability

The datasets generated and/or analyzed during the current study are available from the WEQAA Center Laboratory, Jazan, Saudi Arabia. Requests for access to the data should be directed to Ommer Dafalla (email: omerosa@yahoo.com).

## Supplementary Materials

The following supporting information can be downloaded at: https://www.mdpi.com/article/10.3390/pathogens15030264/s1.

## Abbreviations

RVFV	Rift Valley fever virus
RPMI	Roswell Park Memorial Institute
O.D	optical density
*S*/*N*	signal-to-noise ratio
CPE	cytopathic effect
*TCID*_50_	50% tissue culture infective dose
I	interpolated value of the 50% endpoint
*PD*	proportional distance
VNT	virus neutralization test
C1	initial concentration
C2	final concentration
V1	volume taken from the stock (initial concentration)
V2	final volume
Vero cell	African Green Monkey Kidney Cell Line
FBS	fetal bovine serum
PBS	phosphate-buffered saline
PCT	percentage cumulative technique
