# Supplementary material for: Integrated Laboratory Evaluation of Rift Valley Fever Virus Antibodies Using the Competitive ELISA and Virus Neutralization Test
Source: Pathogens. 2026 Mar 2;15(3):264. doi: 10.3390/pathogens15030264 (PMC13029198; doi:10.3390/pathogens15030264)
Supplement: Supplementary file 1 [file pathogens-15-00264-s001.zip › pathogens-4127991-supplementary.pdf]

Time	virus concentrations		Serum Dilutions (Antibodies titration)										Positive control
			1 : 2	1 : 4	1 : 8	1 : 16	1 : 32	1 : 64	1 : 128	1 : 256	1 : 512	1 : 1024	
			CPE Percentage and number of positive wells										
24H	100000TCID50/0.1ml	CPE Range %	0	0	0	0	0 - 1	0 - 1	0 - 1	0 - 1	10 - 20	10 - 20	10 - 20
		CPE Mean %	0	0	0	0	0.5	0.5	0.5	0.5	15	15	15
		No of Positive Well	4 wells	4 wells	4 wells	4 wells	2 wells	4 wells	4 wells	4 wells	4 wells	4 wells	4 wells
48H	100000TCID50/0.1ml	CPE Range %	0	0 - 1	10 - 20	10 - 20	20 -30	20 - 50	40 - 60	40 - 60	40 - 60	60 - 80	50 - 80
		CPE Mean %	0	0.5	15	15	25	35	50	50	50	70	65
		No of Positive Well	4 wells	1 well	3 wells	4 wells	4 wells	4 wells	4 wells	4 wells	4 wells	4 wells	4 wells
72H	100000TCID50/0.1ml	CPE Range %	0 - 1	1 - 5	20 - 30	40 - 60	70 - 90	70 - 90	90 - 100	100	100	100	100
		CPE Mean %	0.5	3	25	50	80	80	95	100	100	100	100
		No of Positive Well	3 wells	4 wells	4 wells	4 wells	4 wells	4 wells	4 wells	4 wells	4 wells	4 wells	4 wells
96H	100000TCID50/0.1ml	CPE Range %	5 - 15	10 - 20	40 - 50	100	100	100	100	100	100	100	100
		CPE Mean %	10	25	45	100	100	100	100	100	100	100	100
		Protection rate%	90	75	55	0	0	0	0	0	0	0	
		No of Positive Well	4 wells	4 wells	4 wells	4 wells	4 wells	4 wells	4 wells	4 wells	4 wells	4 wells	4 wells
24H	10000TCID50/0.1ml	CPE Range %	0	0	0	0	0	0 - 1	0 - 1	0 - 1	0 - 1	0 - 1	10 - 20
		CPE Mean %	0	0	0	0	0	0.5	0.5	0.5	0.5	0.5	15
		No of Positive Well	4 wells	4 wells	4 wells	4 wells	4 wells	1 well	3 wells	4 wells	4 wells	4 wells	8 wells
48H	10000TCID50/0.1ml	CPE Range %	0	0	0	0 - 1	0 - 1	1 - 5	1 - 5	5 - 10	5 - 10	10 - 20	30-50
		CPE Mean %	0	0	0	0.5	0.5	3	3	7.5	7.5	15	40
		No of Positive Well	4 wells	4 wells	4 wells	1 well	3 well	4 wells	4 wells	4 wells	4 wells	4 wells	4 wells
72H	10000TCID50/0.1ml	CPE Range %	0	0 - 1	0 - 1	0 - 1	1 - 5	5 - 20	20 - 30	40 - 50	50 - 70	80	70 - 80
		CPE Mean %	0	0.5	0.5	0.5	3	12.5	25	45	60	80	75
		No of Positive Well	4 wells	2 wells	3 wells	4 wells	4 wells	4 wells	4 wells	4 wells	4 wells	4 wells	4 wells
96H	10000TCID50/ml	CPE Range %	1 - 5	1 - 10	5 - 15	10 - 20	40 - 80	70 - 100	100	100	100	100	100
		CPE Mean %	3	5.5	10	15	60	85	100	100	100	100	100
		Protection rate%	97	94.5	90	85	40	15	0	0	0	0	
		No of Positive Well	4 wells	4 wells	4 wells	4 wells	4 wells	4 wells	4 wells	4 wells	4 wells	4 wells	4 wells
24H	1000TCID50/0.1ml	CPE Range %	0	0	0	0	0	0	0	0	0 - 1	0 - 1	10 - 20
		CPE Mean %	0	0	0	0	0	0	0	0	0.5	0.5	15
		No of Positive Well	4 wells	4 wells	4 wells	4 wells	4 wells	4 wells	4 wells	4 wells	2 wells	2 wells	4 wells
48H	1000TCID50/0.1ml	CPE Range %	0	0	0	0	0	0	0 - 1	0 - 1	0 - 1	0 - 1	20 - 40
		CPE Mean %	0	0	0	0	0	0	0.5	0.5	0.5	0.5	30
		No of +ve well	4 wells	4 wells	4 wells	4 wells	4 wells	4 wells	3 wells	3 wells	3 wells	3 wells	4 wells
72H	1000TCID50/0.1ml	CPE Range %	0	0	0	0	0	0 - 1	0 - 1	1 - 5	10 - 20	10 - 30	50 - 70
		CPE Mean %	0	0	0	0	0	0.5	0.5	3	15	20	60
		No of Positive Well	4 wells	4 wells	4 wells	4 wells	4 wells	3 wells	3 wells	4 wells	4 wells	4 wells	4 wells
96H	1000TCID50/0.1ml	CPE Range %	1 - 5	1 - 10	5 - 10	5 - 15	10 - 15	15 - 20	20 - 40	20 - 40	70 - 100	70 - 100	90 - 100
		CPE Mean %	3	5.5	7.5	10	12.5	17.5	30	30	85	85	95
		Protection rate%	97	94	92	90	87	82	69	69	11	11	
		No of +ve well	4 wells	4 wells	4 wells	4 wells	4 wells	4 wells	4 wells	4 wells	4 wells	4 wells	4 wells
24H	100TCID50/0.1ml	CPE Range %	0	0	0	0	0	0	0	0	0	0	1 - 5
		CPE Mean %	0	0	0	0	0	0	0	0	0	0	2.5
		No of Positive Well	4 wells	4 wells	4 wells	4 wells	4 wells	4 wells	4 wells	4 wells	4 wells	4 wells	8 wells
48H	100TCID50/0.1ml	CPE Range %	0	0	0	0	0	0	0 - 1	0 - 1	0 - 1	0 - 1	10 - 30
		CPE Mean %	0	0	0	0	0	0	0.5	0.5	0.5	0.5	20
		No of Positive Well	4 wells	4 wells	4 wells	4 wells	4 wells	4 wells	1 well	1 well	1 well	1 well	4 wells
72H	100TCID50/0.1ml	CPE Range %	0	0	0	0	0	0	0 - 1	0 - 1	0 - 1	0 - 1	40 - 50
		CPE Mean %	0	0	0	0	0	0	0.5	0.5	0.5	0.5	45
		No of +ve well	4 wells	4 wells	4 wells	4 wells	4 wells	4 wells	2 wells	2 wells	2 wells	2 wells	4 wells
96H	100TCID50/0.1ml	CPE Range %	1 - 5	1 - 5	5 - 15	5 - 15	5 - 15	10 - 20	10 - 20	10 - 20	10 - 30	10 - 30	70 - 80
		CPE Mean %	3	3	10	10	10	15	15	15	20	20	75
		Protection rate%	96	96	87	87	87	80	80	80	74	74	
		No of Positive Well	4 wells	4 wells	4 wells	4 wells	4 wells	4 wells	4 wells	4 wells	4 wells	4 wells	4 wells
